# Clinical outcomes of temporal bone squamous cell carcinoma: A single‐institution experience

**DOI:** 10.1002/cam4.5338

**Published:** 2022-10-13

**Authors:** Yongbo Zheng, Ke Qiu, Yangju Fu, Wenjie Yang, Danni Cheng, Yufang Rao, Minzi Mao, Yao Song, Wei Xu, Jianjun Ren, Yu Zhao

**Affiliations:** ^1^ Department of Otorhinolaryngology, Head & Neck Surgery West China Hospital, Sichuan University Chengdu China; ^2^ Operating Room West China Hospital, Sichuan University/West China School of Nursing, Sichuan University Chengdu China; ^3^ Center of Biostatistics, Design, Measurement and Evaluation(CBDME) West China Hospital, Sichuan University Chengdu China; ^4^ Department of Biostatistics Princess Margaret Cancer Centre and Dalla Lana School of Public Health Toronto Ontario Canada

**Keywords:** overall survival, prognostic factor, temporal bone squamous cell carcinoma

## Abstract

**Objectives:**

This study aimed to investigate the survival outcomes and potential prognostic factors of patients with temporal bone squamous cell carcinoma (TBSCC) treated at our institution.

**Methods:**

We retrospectively included patients who were diagnosed with TBSCC between 2008 and 2019. The Kaplan–Meier (KM) method was used to describe overall survival (OS), and the association between baseline characteristics and prognoses was examined using Cox proportional hazards models.

**Results:**

Fifty consecutive patients with TBSCC were included in this study. The results showed that patients with advanced modified Pittsburgh (MPB)‐ T classifications had a poorer prognosis (T3 vs. T1‐2: HR: 2.81, 95% CI: 0.34–23.43; T4 vs. T1‐2: HR: 7.25, 95% CI: 0.95–55.41; *p* = 0.041). Meanwhile, middle ear squamous cell carcinoma (MESCC) showed a significantly worse prognosis than external auditory canal squamous cell carcinoma (EACSCC, HR: 2.65, 95% CI: 1.04–6.76, *p* = 0.04).

**Conclusions:**

MESCC and advanced MPB‐T classifications might be considered predictors of unfavorable outcomes in patients with TBSCC, indicating that special attention should be paid to the original tumor subsite and tumor extension in the management of patients with TBSCC.

## Graphical abstract

The successful treatment of temporal bone squamous cell carcinoma (TBSCC) is complicated by a number of issues originated from its rarity. We retrospectively analyzed 50 TBSCC patients treated in our single institution. Our study implicated that MESCC and advanced MPB (modified Pittsburgh)‐T classifications might be considered as predictors of unfavorable outcomes in TBSCC patients, indicating that special attention should be paid to the original tumor subsite and tumor extension in the management of TBSCC patients.

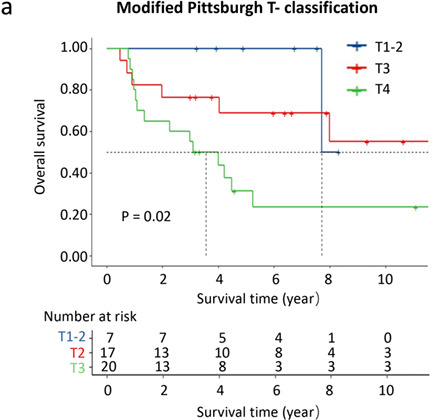

## 1 INTRODUCTION

Temporal bone carcinomas (TBCs) account for a small portion (0.2%) of all malignancies of the head and neck region, with an estimated annual incidence of 1–6 cases per million population.^1^, ^2^ Primary TBCs are highly heterogeneous carcinomas of varying origins, with the external auditory canal (EAC) and middle ear (ME) being the most commonly involved primary sites and squamous cell carcinoma predominating among all histological types.^3^, ^4^

Temporal bone squamous cell carcinoma (TBSCC) typically causes nonspecific symptoms such as otalgia, otorrhea, and hearing loss, making it difficult for clinicians to discriminate TBSCC from inflammatory diseases.^3^ Surgical resection with or without radiotherapy is currently the mainstay of curative treatment for TBSCC, and the choice of treatment mainly depends on the degree of tumor extension. Although some improvements have been achieved in recent decades, the overall survival of patients with TBSCC remains poor.^5^, ^6^

One major obstacle in TBSCC management is the absence of a recognized staging system for TBSCCs, which seriously impedes rational comparison and integration of treatment efficacy between clinical studies. The MPB staging system was first proposed in 2000 and has subsequently gained global popularity in the clinical management of external auditory canal squamous cell carcinoma (EACSCC).^7^ However, its prognostic accuracy for TBSCC of other origins requires further investigation. Additionally, most TBSCC‐related studies have been reported in the form of case series, which mostly included small but highly heterogeneous populations, thereby greatly limiting the comprehensive understanding of potential prognostic factors.^8^, ^9^

Overall, successful treatment of TBSCC is complicated by several issues originating from its rarity. Integration of multi‐institutional clinical outcomes of TBSCC is urgently required and could contribute to a more comprehensive understanding of this rare disease. Therefore, the primary objective of this study was to evaluate the management strategies and survival outcomes of patients with TBSCC treated at our institution. We also aimed to examine the prognostic value of the modified Pittsburgh staging system and to identify other potential prognostic factors for TBSCC.

## 2 PATIENTS AND METHODS

### 2.1 Eligibility criteria

Patients with pathologically confirmed primary TBSCC diagnosed at West China Hospital from August 2008 to September 2019 were retrospectively included in this study. Cases that met the following criteria were excluded: (1) the temporal bone was not the primary site of involvement; (2) pathological results did not indicate squamous cell carcinoma; and (3) follow‐up information was incomplete. The study protocol was approved by the Biomedical Research Ethics Committee of West China Hospital (approval number: 2019–357), and the requirement for informed consent was waived because of the retrospective nature of the study.

### 2.2 Data extraction and staging strategy

Data for the patients' age, sex, marital status, race, smoking history and pack‐years, alcohol consumption, tumor subsites and extent, histologic type, treatment modalities, including surgery, radiotherapy and chemotherapy, comorbidities, and onset of symptoms were reviewed. Patients were retrospectively staged using the MPB^7^ staging system by two experienced otologists according to the pathological results, imaging, and operative findings. Discrepancies were resolved by consensus.

### 2.3 Treatment modalities

The management strategies for all patients were discussed by a multidisciplinary team. Lateral temporal bone resection was performed in patients with early stage (T1‐T2) TBSCC, whereas patients with more locally advanced (T3‐T4) tumors were treated using lateral temporal bone resection or individualized subtotal temporal bone resection based on the extent of the disease. Patients with margin positivity, nodal metastasis, or extracapsular extension were treated with adjuvant therapy. Discrepancies were resolved through consensus. The extent of resection was determined preoperatively by the operating surgeon and further customized based on intraoperative findings and frozen sections.

### 2.4 Follow‐up

Follow‐up assessments were conducted annually, in accordance with the surveillance guidelines at our center. The date of death was extracted, and overall survival rates were calculated accordingly. Due to data limitations, recurrence‐free and disease‐specific survival could not be investigated.

### 2.5 Statistical analysis

Continuous variables are presented as means and standard derivations, as well as medians and ranges, whereas categorical variables are presented as frequencies and percentages. Kruskal–Wallis tests were performed to compare continuous variables, whereas Fisher's exact tests were performed for categorical variables. The Kaplan–Meier (KM) method was used to describe overall survival (OS), and the association between baseline characteristics and prognoses was examined using Cox proportional hazards models. Two‐tailed tests were used, and P values less than 0.05 were considered significant. The variables adjusted in the multivariable analysis were selected according to the results of the univariate analysis and clinical significance. All data were analyzed using R software (R Foundation, version 3.1.2).

## 3 RESULTS

### 3.1 Baseline characteristics of all patients with TBSCC

Fifty patients with a mean age of 60 years were included in this study. The EAC was the most commonly involved primary subsite (46%), followed by the ME (38%). Approximately 45% of these patients were categorized under T4, followed by T3 (39%), and T1‐2 (16%). Node metastasis was observed in 18% of the patients, while only 2% of the patients showed progression to distant metastasis. Overall, nearly half of the patients had stage IV disease, followed by stage III (32%), stage I (9%), and stage II (5%). Approximately half of the patients underwent surgery alone after diagnosis, while 24% received postoperative adjuvant therapy. Facial paralysis was only observed in 12% of the patients. Detailed baseline information is presented in Table 1.

**TABLE 1:** Baseline characteristics of TBSCC patients (full sample size, *n* = 50)

Covariate	Number of patients (*n* = 50)
Gender	
Female	12 (24)
Male	38 (76)
Age	
Mean ± SD	60 ± 12
Median (range)	61 (30–79)
Smoking history	
Ever	25 (52)
Never	23 (48)
Missing	2
Pack year	
≥30	18 (38)
<30	30 (62)
Missing	2
Alcohol consumption	
Never	21 (45)
Ever	26 (55)
Missing	3
Subsite	
EAC	23 (46)
ME	19 (38)
Mastoid	8 (16)
T classification	
T1‐2	7 (16)
T3	17 (39)
T4	20 (45)
Missing	6
N classification	
N0	36 (82)
N1‐3	8 (18)
Missing	6
M classification	
M0	43 (98)
M1	1 (2)
Missing	6
Overall stage	
I	4 (9)
II	2 (5)
III	14 (32)
IV	24 (55)
Missing	6
Treatment modality	
Other	12 (24)
Surgery only	26 (52)
Surgery + RT/CT/RCT	12 (24)
Radiotherapy	
Yes	14 (33)
No	29 (67)
Missing	7
Chemotherapy	
Yes	5 (12)
No	38 (88)
Missing	7
Surgery	
Yes	44 (88)
No	6 (12)

Abbreviations: CT, chemotherapy; EAC, external auditory canal; ME, middle ear; RCT, chemoradiation; RT, radiotherapy.

As shown in Table 2, patients who underwent surgery plus postoperative adjuvant therapy had significantly better survival than those who underwent surgery alone (5‐year OS: 83% vs. 45%). As expected, a gradual decline in survival rates was observed in patients as the T classification increased (5‐year OS: T1‐2: 100%; T3: 69%; T4: 31%; *p* = 0.02). Meanwhile, patients with middle ear squamous cell carcinoma (MESCC) had significantly lower survival rates than patients with EACSCC (5‐year OS: 37% vs. 78%, *p* < 0.001).

**TABLE 2:** 1‐year, 3‐year and 5‐year overall survival rates of TBSCC patients based on various covariates

Covariate	Strata	Overall survival (%)				
		Events/Total	1‐year (95% CI)	3‐year (95% CI)	5‐year (95% CI)	*p*‐value
Gender	Male	19/38	87 (77–98)	74 (61–89)	57 (42–76)	0.40
	Female	5/12	83 (65–100)	67 (45–100)	67 (14–100)	
Smoking history	Ever	11/25	92 (82–100)	76 (61–95)	59 (40–85)	1.00
	Never	12/23	83 (69–100)	70 (53–91)	60 (43–84)	
Pack year	≥30	9/18	89 (76–100)	72 (54–96)	58 (38–88)	0.60
	<30	14/30	87 (75–100)	73 (59–91)	61 (45–83)	
Alcohol consumption	Ever	11/21	91 (79–100)	76 (60–97)	54 (35–82)	0.50
	Never	12/26	85 (72–100)	69 (54–90)	64 (48–86)	
Treatment modality	Other	5/12	83 (65–100)	67 (45–100)	67 (45–100)	0.09
	Surgery	17/26	85 (72–100)	69 (54–90)	45 (29–72)	
	Surgery + RT/CT/RCT	2/12	92 (77–100)	83 (63–100)	83 (63–100)	
Radiotherapy	Yes	4/14	93 (80–100)	78 (59–100)	78 (59–100)	0.05
	No	20/29	79 (66–96)	62 (47–83)	41 (25–65)	
Chemotherapy	Yes	2/5	100	80 (52–100)	80 (52–100)	0.20
	No	22/38	82 (70–95)	66 (52–83)	47 (32–69)	
T classification	T1‐2	1/7	100	100	100	0.02*
	T3	7/17	82 (66–100)	77 (59–100)	69 (49–96)	
	T4	14/20	80 (64–100)	55 (37–82)	31 (16–63)	
N classification	N0	17/36	81 (69–95)	67 (53–84)	56 (41–76)	0.80
	N1‐3	5/8	100	88 (67–100)	60 (33–100)	
M classification	M0	21/43	84 (73–96)	70 (57–85)	58 (45–76)	0.50
	M1	1/1	100	100	0	
Overall stage	I	0/4	100	100	100	0.08
	II	0/2	100	100	100	
	III	6/14	86 (69–100)	79 (60–100)	69 (47–100)	
	IV	16/24	79 (65–97)	58 (42–82)	40 (24–66)	
Facial paralysis	Yes	4/6	67 (38–100)	50 (11–100)	33 (11–100)	0.20
	No	20/44	89 (80–99)	75 (63–89)	62 (49–80)	
Subsite	EAC	8/23	100	78 (62–97)	78 (62–97)	0.10
	ME	12/19	68 (50–93)	58 (40–85)	37 (20–66)	
	Mastoid	4/8	88 (67–100)	88 (67–100)	73 (47–100)	

Abbreviations: CT, chemotherapy; EAC, external auditory canal; ME, middle ear; RCT, chemoradiation; RT, radiotherapy; SCC, squamous cell carcinoma.

* *p* < 0.05.

### 3.2 Univariate analysis in all patients with TBSCC

**FIGURE 1:**
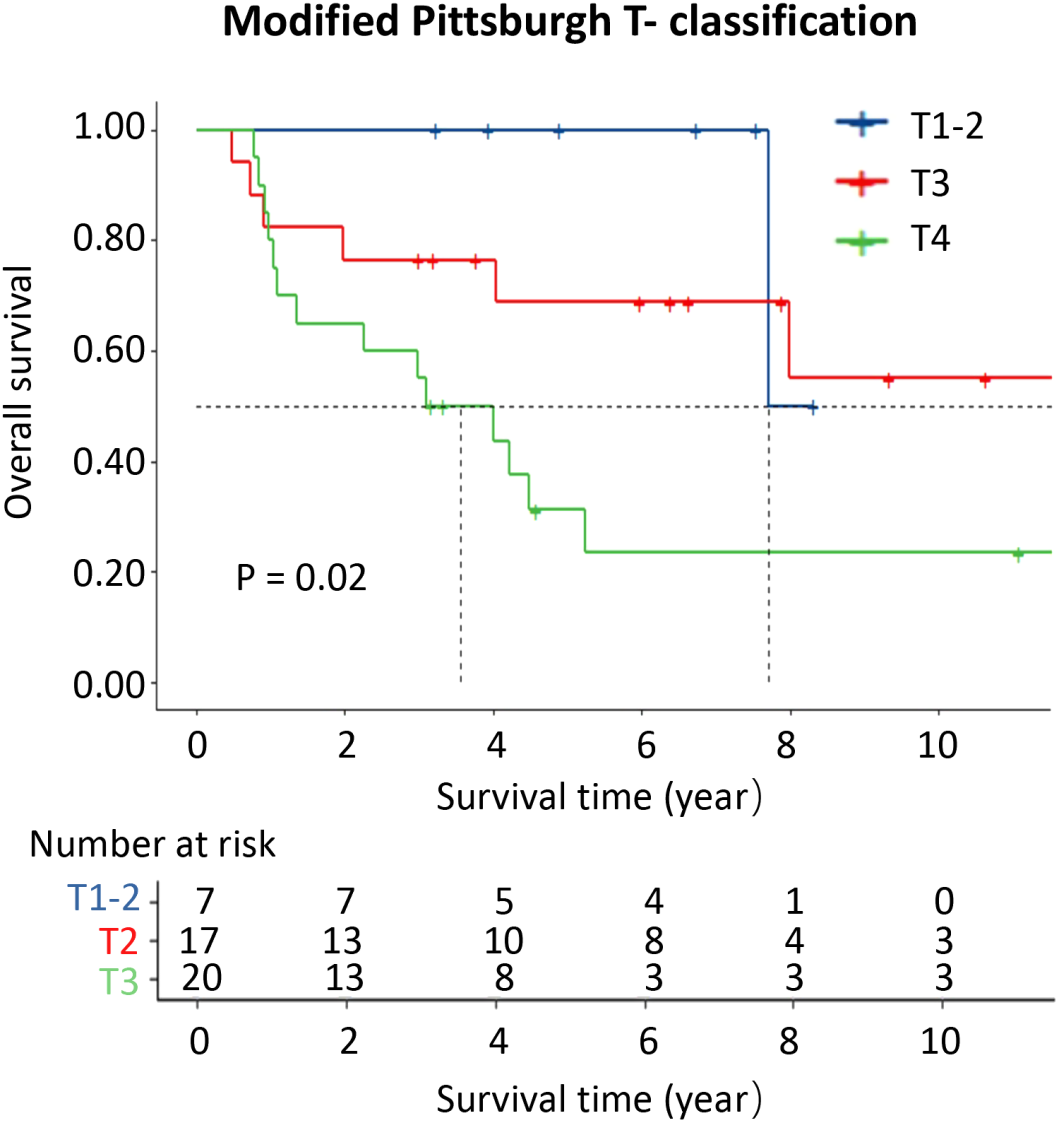
KM curves of overall survival grouped by Modified Pittsburgh T‐ classification

Univariate analysis showed that MESCC (*p* = 0.04) and advanced MPB‐T classification (*p* = 0.041) were predictors of poor prognosis in patients with TBSCC (Table 3). Specifically, advanced MPB‐T classification was associated with poor prognosis (T3 vs. T1‐2: HR: 2.81, 95% CI: 0.34–23.43; T4 vs. T1‐2: HR: 7.25, 95% CI: 0.95–55.41, *p* = 0.041, Figure 1). Notably, MESCC showed a significantly worse prognosis than EACSCC (HR, 2.65; 95% CI: 1.04–6.76, *p* = 0.04).

**TABLE 3:** Univariable cox model for overall survival of TBSCC patients (*n* = 50)

Covariate	HR (95% CI)	*p*‐value	Global *p*‐value Likelihood ratio test
Age (continuous)	1.01 (0.97,1.05)	0.62	0.62
Gender			0.38
Male	Reference		
Female	0.61 (0.21,1.81)	0.38	
Smoking history			0.95
Ever	Reference		
Never	0.98 (0.42,2.28)	0.95	
Pack year			0.56
≥30	Reference		
<30	0.77 (0.33,1.83)	0.56	
Alcohol consumption			0.47
Ever	Reference		
Never	0.73 (0.31,1.71)	0.47	
Treatment modality			0.12
Surgery only	Reference		
Surgery + RT/CT/RCT	0.57 (0.21,1.57)	0.28	
Other	0.24 (0.05,1.03)	0.06	
Radiotherapy			0.06
Yes	Reference		
No	2.86 (0.97,8.43)	0.06	
Chemotherapy			0.26
Yes	Reference		
No	2.33 (0.54,10.09)	0.26	
Overall stage			0.54
I	NA		
II	NA		
III	0.47 (0.17–1.29)	0.10	
IV	Reference		
T classification			*0.041* *
T1‐2	Reference		
T3	2.81 (0.34,23.43)	0.34	
T4	7.25 (0.95,55.41)	0.06	
N classification			0.79
N0	Reference		
N1‐3	1.15 (0.42,3.14)	0.79	
Subsite			0.11
EAC	Reference		
ME	2.65 (1.04,6.76)	0.04*	
Mastoid	1.41 (0.41,4.83)	0.59	
Facial paralysis			0.18
Yes	Reference		
No	0.48 (0.16,1.41)	0.18	

Abbrevistions: CT, chemotherapy; EAC, external auditory canal; ME, middle ear; RCT, chemoradiation; RT, radiotherapy; SCC, squamous cell carcinoma.

* *p* < 0.05.

### 3.3 Multivariate analysis in all patients with TBSCC

After adjusting for age, subsite, and treatment modality, although patients with advanced MPB‐T classification showed obviously worse prognosis (T3 vs. T1‐2: HR: 2.24, 95% CI: 0.25–20.05; T4 vs. T1‐2: HR: 5.36, 95% CI: 0.60–47.91), MPB‐T classification only reached a marginal significance for prognosis prediction (*p* = 0.06) (Table 4).

**TABLE 4:** Multivariable models of overall survival in TBSCC patients based on T classification

Covariate	Model 1			Model 2		
	HR (95% CI)	*p*‐value	Global *p*‐value	HR (95% CI)	*p*‐value	Global *p*‐value
T classification			0.05			0.06
T1‐2	Reference			Reference		
T3	2.55 (0.27–23.74)	0.41		2.24 (0.25–20.05)	0.47	
T4	6.18 (0.68–56.16)	0.11		5.36 (0.60–47.91)	0.13	

Abbreviations: CT, chemotherapy; RCT, chemoradiation; RT, radiotherapy.

Model 1 was adjusted for age and subsite, Model 2 was adjusted for age, subsite and treatment modality.

## 4 DISCUSSION

In the present study, we comprehensively investigated the survival outcomes and potential prognostic factors of patients with TBSCC treated in our institution. We confirmed that MESCC and advanced MPB‐T classification were predictors of poor prognosis in patients with TBSCC.

A uniform staging system for TBSCC is still lacking, forcing clinicians to continue exploring novel staging schemes and investigating their scope of application. Stell et al. first proposed the Stell‐T classification in 1985, which was derived from an analysis of a small number of heterogeneous patients with TBSCC in a single institute. Thus, generalization of these results to the entire TBC population may yield inadequate accuracy and reliability.^10^ Similarly, another well‐known staging system, the MPB staging system (derived from EACSCC), has also been investigated as a significant prognostic factor for patients with TBSCC by numerous studies.^6^, ^8^, ^9^, ^11^, ^12^, ^13^, ^14^, ^15^, ^16^, ^17^, ^18^ Nevertheless, most of these studies only confirmed the MPB‐T classification as a significant prognostic factor rather than the overall stage, which is consistent with our results. Meanwhile, the majority of the above‐mentioned studies included a heterogeneous population of patients with TBSCC, which mainly consisted of patients with EACSCC; thus, the validity of the MPB‐T classifications for MESCC remains unclear. Notably, several studies have also confirmed the prognostic value of the MPB‐T classification in other histological types of TBC, suggesting that its application range might be wider than previously thought.^19^, ^20^

The American Joint Committee on Cancer (AJCC) staging system is the most widely applied staging guideline for squamous cell carcinomas originating from the head and neck region. However, it is considered inappropriate for TBSCC, mainly because of the highly specific features of this region, which is characterized by the integration of various sophisticated anatomical structures in a very limited space. Zanoletti et al. compared the prognostic performance of the 8th AJCC staging system and the MPB staging system, and the results showed that the prognostic performance was only acceptable for both of them.^21^ Therefore, further exploration of new staging systems is urgently required.

Additionally, our results also demonstrated that MESCC showed a worse prognosis than TBSCC originating from other subsites, which is consistent with the findings of previous studies,^22^, ^23^, ^24^ indicating the need to pay special attention to the original tumor subsite, an important confounding factor, while addressing TBSCC‐related issues. Notably, Komune et al. demonstrated a high proportion of tumor extension to ME cavity in primary TBSCC not originating from the ME, which was further confirmed to be not associated with poorer prognosis.^12^ In combination with our results, these findings indicate that the ME as the primary site rather than secondarily involved sites might be a predictor of poor prognosis in patients with TBSCC.

The main strength of this study is that we paid special attention to the MPB‐T classification and confirmed its prognostic significance for overall survival of patients with TBSCC, providing evidence for its further application as well as revision. Nevertheless, our study also had several limitations. First, we included a mixed population of TBSCC composed of EAC and ME, and the small sample size prevented us from obtaining more definitive conclusions through a subgroup analysis based on the subsite. Therefore, our results must be interpreted with caution considering EACSCC and MESCC together. In addition, after adjustment for age, subsite, and treatment modality, the MPB‐T classification lost its statistical significance for predicting the prognosis of patients with TBSCC, which could be attributed to the small sample sizes and the potential bias from mixed tumor subsites. Therefore, the prognostic value of the MPB‐T classification remains to be validated in further multicenter studies with large sample sizes.

## 5 CONCLUSIONS

Overall, MESCC and advanced MPB‐T classifications can be considered to be predictors of unfavorable outcomes in patients with TBSCC, indicating that special attention should be paid to the original tumor subsite and tumor extension in the management of patients with TBSCC. In addition, MESCC and TBSCC originating from other subsites should be analyzed separately instead of combining them, especially when using the modified Pittsburgh classification. A proper staging scheme specifically designed for MESCC is also required.

## AUTHOR CONTRIBUTIONS

**Yongbo Zheng:** Conceptualization (lead); formal analysis (equal); investigation (lead); methodology (equal); project administration (equal); writing – original draft (lead); writing – review and editing (lead). **Ke Qiu:** Conceptualization (equal); investigation (lead); methodology (equal); writing – original draft (equal). **Yangju Fu:** Data curation (equal); investigation (equal); writing – original draft (equal); writing – review and editing (equal). **Wenjie Yang:** Formal analysis (lead); investigation (equal); methodology (lead); writing – original draft (equal). **Danni Cheng:** Data curation (equal); investigation (equal). **Minzi Mao:** Investigation (equal). **Yufang Rao:** Data curation (equal). **Yao Song:** Formal analysis (equal). **Wei Xu:** Conceptualization (equal); methodology (lead); project administration (equal); writing – review and editing (equal). **Jianjun Ren:** Conceptualization (lead); project administration (lead); writing – review and editing (equal). **Yu Zhao:** Conceptualization (lead); funding acquisition (lead); project administration (lead); writing – review and editing (equal).

## FUNDING INFORMATION

This work was supported by the [The Science and Technology Department of Sichuan Province] under Grant [number 2022YFS0066]; [China Postdoctoral Science Foundation] under Grant [number 2020M673250]; [National Natural Youth Science Foundation of China] under Grant [number 82002868].

## CONFLICT OF INTEREST

The Authors declare that there is no conflict of interest.

## ETHICAL APPROVAL STATEMENT

The study protocol was approved by the Biomedical Research Ethics Committee of West China Hospital (approval number: 2019–357) and informed consent was waived because of its retrospective nature.

## Data Availability

The data that support the findings of this study are available from the corresponding author upon reasonable request.
